# Understanding how context and culture in six communities can shape implementation of a complex intervention: a comparative case study

**DOI:** 10.1186/s12913-022-07615-0

**Published:** 2022-02-17

**Authors:** Jessica Gaber, Julie Datta, Rebecca Clark, Larkin Lamarche, Fiona Parascandalo, Stephanie Di Pelino, Pamela Forsyth, Doug Oliver, Dee Mangin, David Price

**Affiliations:** Department of Family Medicine, McMaster University, David Braley Health Sciences Centre, 100 Main Street West, Hamilton, Ontario L8P 1H6 Canada

**Keywords:** Case study, Primary care, Interprofessional health care teams, Volunteers, Qualitative research

## Abstract

**Background:**

Contextual factors can act as barriers or facilitators to scaling-up health care interventions, but there is limited understanding of how context and local culture can lead to differences in implementation of complex interventions with multiple stakeholder groups. This study aimed to explore and describe the nature of and differences between communities implementing Health TAPESTRY, a complex primary care intervention aiming to keep older adults healthier in their homes for longer, as it was scaled beyond its initial effectiveness trial.

**Methods:**

We conducted a comparative case study with six communities in Ontario, Canada implementing Health TAPESTRY. We focused on differences between three key elements: interprofessional primary care teams, volunteer program coordination, and the client experience. Sources of data included semi-structured focus groups and interviews. Data were analyzed through the steps of thematic analysis. We then created matrices in NVivo by splitting the qualitative data by community and comparing across the key elements of the Health TAPESTRY intervention.

**Results:**

Overall 135 people participated (39 clients, 8 clinical managers, 59 health providers, 6 volunteer coordinators, and 23 volunteers). The six communities had differences in size and composition of both their primary care practices and communities, and how the volunteer program and Health TAPESTRY were implemented. Distinctions between communities relating to the work of the interprofessional teams included characteristics of the huddle lead, involvement of physicians and the volunteer coordinator, and clarity of providers’ role with Health TAPESTRY. Key differences between communities relating to volunteer program coordination included the relationship between the volunteers and primary care practices, volunteer coordinator characteristics, volunteer training, and connections with the community. Differences regarding the client experience between communities included differing approaches used in implementation, such as recruitment methods.

**Conclusions:**

Although all six communities had the same key program elements, implementation differed community-by-community. Key aspects that seemed to lead to differences across categories included the size and spread of communities, size of primary care practices, and linkages between program elements. We suggest future programs engaging stakeholders from the beginning and provide clear roles; target the most appropriate clients; and consider the size of communities and practices in implementation.

**Trial registration:**

ClinicalTrials.gov: NCT03397836.

**Supplementary Information:**

The online version contains supplementary material available at 10.1186/s12913-022-07615-0.

## Background

After effectiveness and feasibility are demonstrated in initial evaluations of new health interventions, interventions often undergo further trials in new or expanded contexts. This long but necessary process, referred to as scaling-up, contributes to the development of sustainable, relevant, and effective interventions [1] and can improve the external validity or generalizability of the intervention. However, interventions often have difficulties retaining their effectiveness compared to the original trial when the implementation is expanded out to new settings [2].

An extensive list of intertwined constructs that influence program implementation means that complex interventions rarely function the same way in different contexts [3, 4]. These constructs include the nature of the intervention; the target population; the organization implementing the intervention (e.g., organizational culture); the available resources (e.g., time, physical space, staffing); and the individuals within the organization (e.g., buy-in of personnel delivering the intervention, perceptions of key stakeholders) [4]. Factors within each of these constructs can be categorized as barriers or facilitators to successfully scaling up interventions, i.e., being able to conduct the intervention at all, and the potential for replicating results. For example, commonly cited barriers include a lack of resources in the new setting, staff turnover, or leadership changes during implementation [1].

In this paper, we discuss Health TAPESTRY [5], a primary care-based intervention. Community volunteers are included to help interprofessional primary care teams in supporting older adult clients in staying healthier at home for longer. Technology facilitates the program, and community engagement and connections are used to help fulfill clients’ health-related social needs. In the initial implementation of Health TAPESTRY, a randomized controlled trial completed in one primary care practice, the results indicated that the intervention group had fewer hospitalizations, had more primary care visits, and walked more compared to the control group at a 6-month follow-up [6].

A key element of Health TAPESTRY is that there are multiple groups involved in the program. Health care providers are responsible for the collective creation and implementation of the clients’ plans of care. Collaborative teamwork in primary care is not guaranteed even if the health care providers work in the same space as teamwork is facilitated by effective communication, a shared understanding of roles, trust, respect, and regular interactions with each other [7–11]. Commonly reported barriers to interprofessional teamwork include lack of role clarity, time, resources, communication, and physical space [8, 10, 11]. The second key stakeholder group in Health TAPESTRY are the volunteers. Good program coordination is important to keep volunteers; volunteers who experience issues with program administration, or have issues with work environments or staff feel dissatisfied [12, 13], whereas volunteer programs that provide good training, feedback and opportunities for volunteers to express themselves, are viewed positively [12, 14–16]. The third key stakeholder group are clients of the program. How well clients engage with a program is very important to understand. Feedback and input from clients help ensure new programs are applicable and beneficial to the target population and feasible overall although literature specifically focused on patient experience in complex health interventions is relatively limited [17, 18].

While other studies or reports have looked at contextual factors, barriers, and facilitators to scaling up health interventions, this information is most often summarized across the entire program, and rarely examined for differences between implementation communities [1, 4, 19–22]. Exploring key contextual factors and how they are connected within each community could provide insight into the specific mechanisms that facilitate the implementation of complex interventions while accounting for communities’ unique attributes. The complexity of the Health TAPESTRY intervention, with its multiple stakeholder groups who work together in a coordinated effort to provide care to patients, is also rarely seen in the literature, and could fill a gap of providing multiple perspectives. The objective of this comparative case study is to explore and describe the nature of and differences between six communities as they implement Health TAPESTRY, specifically with respect to three key areas of difference between them: 1) interprofessional teams’ patterns of work, 2) volunteer program coordination, and 3) the client experience.

## Methods

### Setting

The implementation of Health TAPESTRY was facilitated by an academic family medicine department and conducted within six communities of varying sizes across Ontario, Canada. This implementation of the program was a scaling-up of the earlier implementation of Health TAPESTRY during the initial effectiveness trial [6]. Each community was associated with a family health team (FHT). FHTs are physician-led primary care practices with embedded interprofessional care providers and are a common model for providing primary care in the province [23]. As the intervention also included community volunteers conducting home visits to older adult clients, the setting also included client homes in the six communities, as well as the organizations that coordinated the volunteers’ work. Four of the communities were supported by a national humanitarian charitable organization while the other two were supported by a coalition of agencies focused on community health.

### Health TAPESTRY

In Health TAPESTRY, two trained volunteers visit older adult clients in their homes and use structured surveys to ask about clients’ health needs, goals, and social context [5]. Client responses are entered into a web-based application (the TAP-App) using a tablet computer. A summary (the TAP-Report) is sent to the client’s primary care team where a plan of care is created and implemented by a huddle team, a small group of interprofessional health care team members. After six months, the volunteers return to clients’ homes to repeat the surveys and determine if clients’ goals were met. Each community has a huddle lead who facilitates the huddle and a physician champion who is an advocate for the program and actively involved. FHTs can choose to provide clients with a client-friendly TAP-Report which is a brief summary of their survey results and a written explanation of the plan of care. They can also choose to send volunteers back within the six-months to do any necessary follow-up.

### Design and definitions

This qualitative case study takes inspiration in its design from Bartlett and Vavrus’s comparative case study (CCS) methodology. CCS describes culture and context more broadly than in many case study methodologies and we will use those definitions in this paper. According to CCS, culture can be defined as *the development of sense-making processes* rather than a single static ‘culture’; context can be understood beyond geographical boundaries or a strictly bounded case and defined more fully as *interconnectedness with surroundings and hierarchies* [24]. Specifically in our project, when we talk about culture we are talking about the personal, relationship-based, and organizational culture of the individuals involved in implementing Health TAPESTRY and context as interconnectedness between these people, their organizations, and beyond.

We compared our six cases (the six Ontario communities implementing Health TAPESTRY) using a process-oriented approach to make sense of implementation [24]. When we talk about implementation in this paper, we mean how communities managed to carry out (i.e., implement) the Health TAPESTRY intervention. To understand this concept, we focused on perspectives of our multiple stakeholder groups on what is working well (i.e., facilitators of implementation) and what is not working well (i.e., barriers to implementation). We also incorporated considerations of power structures and relations, horizontal comparisons between communities, and vertical and longitudinal feedback about hierarchy and context where available [24]. In this paper, we chose to focus on aspects of the cases that had distinctions between communities, rather than those that were common between them.

### Data collection and participants

Focus groups, interviews, and narratives from key informants on the research team were used to describe and compare the cases. We conducted separate focus groups in each community for each of three stakeholder groups (members of the huddle team, providers outside the huddle, and volunteers). We conducted semi-structured interviews with clients, clinic managers, volunteer coordinators, and other key providers or volunteers who could not make the focus group time. All members involved with Health TAPESTRY (listed above) in each site were invited to participate in either a focus group or interview by email, except for the clients. We used convenience sampling stratified by site to invite intervention clients by telephone to participate in an interview. Implementation was in a rolling fashion; however, data collection across each community had the same timeline: client interviews were conducted once clients had finished their 6-month volunteer visit, volunteer and volunteer coordinator data were collected one year after volunteers had first visited clients in that community, and clinic team members’ data were collected after ten 6-month reports had been seen in that community. All focus groups were held in the participants’ communities at a primary care clinic (with at least one of the two facilitators in person), and most interviews were held over the phone, with a few in person if a facilitator was visiting the participant’s site (e.g., with some clinical managers or volunteer coordinators).

Focus groups were facilitated at a community location by two research team members, at least one of whom was in-person with participants. Interviews had one facilitator and were either in person or over the phone, depending on participant availability and convenience. Facilitators included: HB, RC, JD, SD, JG, CK, and FP. The focus groups and interviews were semi-structured, using a question guide informed by the interview guide from the previous implementation of Health TAPESTRY, and slightly adapted for each participant group, with a focus on program improvement. Interviews and focus groups were audio recorded and transcribed. Facilitators also made field notes.

Key implementers of Health TAPESTRY in the research team reviewed the data that were collected and added an understanding of the implementation across the six communities including the context, culture, and hierarchies inherent in each case (i.e., community).

### Data analysis

Transcripts of focus groups and interviews were uploaded into NVivo 12 [25]. Three researchers with experience in qualitative research and Health TAPESTRY (SD, JG, and FP) coded and analyzed the transcripts. The three coders all identify as female, have graduate degrees, may have known some of the participants in the interviews and focus groups, and had comprehensive knowledge about Health TAPESTRY as they were both implementors and evaluators of the program. We followed the six phases of thematic analysis described in Braun and Clarke [26, 27] as described below, but coded at a more semantic rather than a reflexive or latent level. First, JG and FP independently familiarized themselves with the data (phase 1), and then jointly created a basic coding structure based on our interview questions. Initial transcripts were coded deductively (phase 2) based on the question guide (Additional File 1), i.e., the initial categories within the codebook were based on the key questions we asked in the interviews and focus groups, which in turn were the general areas we wanted to probe in order to understand participants’ perspectives on Health TAPESTRY and its implementation (e.g., what was working well and what was not working well with the program). This was based on our previous program implementation and our program evaluation needs in this round. The remaining coding was generated more inductively, adding further codes and categories beyond the basic categories already included. All transcripts were independently coded by one of the coders and then checked by another coder to ensure consistency. We then began searching for (phase 3) and reviewing themes and re-organizing codes into higher level categories and themes (phase 4) and then naming the themes (phase 5). Any disagreements were resolved through regular discussion and meetings between analysts (with the occasional inclusion of RV). Phase 6 of thematic analysis is creating a report; the report is this manuscript.

After the preparation of the NVivo database including focus group and interview data from all sources, we split cases (i.e., the six Ontario communities implementing the program) and compared them on all elements of the Health TAPESTRY model via the matrices function. There is a long history of the use of matrices in qualitative research [28]. Converting the textual qualitative data to numbers, often referred to as “quantitizing” in the mixed methods literature [29–31], can help researchers identify patterns in the data, clarify meaning in the data, contribute to the display of data, and help readers understand and interpret the data [28, 32, 33]. We used intensity matrices, where cell contents are numerical and higher numbers indicate higher intensities of frequencies [28], and we did this on a case-by-variable matrix [29]. Using the matrices produced by NVivo in this way allowed our research team to visualize the proportions of frequencies by community across themes. While the Health TAPESTRY program as a whole has multiple important elements, stakeholders, and constructs, to compare between sites we wanted to compare only the elements that showed differences. JD, JG, and LL reviewed, compared, and discussed the matrices to understand which aspects were distinctive between communities. It was at this point that we determined that while several categories and themes were very similar across communities, three elements had differences between communities: 1) interprofessional teams’ patterns of work, 2) volunteer program coordination, and 3) the client experience.

Once reviewing all matrices, we chose to focus on the description of three key areas of Health TAPESTRY which were the most distinct between communities: the work of interprofessional primary care teams, volunteer program coordination, and the client experience; incidentally, these also represented the three key stakeholder groups in the dataset. The percentages within the matrices are indicative of the frequency of each theme (row) within a category by community. Afterwards, these researchers provided narratives about additional contextual factors that may have led to these differences, as well as potential implications that would not have been shown in the qualitative data alone (Table 2).

Enhancing qualitative rigour was considered in varied ways. Credibility and confirmability were enhanced by using multiple data sources (interviews, focus groups), multiple perspectives (clients, health care team members, volunteer coordinators, and volunteers), and multiple analysts (JD, SD, FP, JG, and LL) [24, 34]. We used the COREQ checklist to guide our reporting of this study (Additional File 2). The potential generation of theoretical insights that could help understand cases beyond the ones described in this paper were enhanced through thick description in settings and cases [24].

### Framework and theoretical background

Health TAPESTRY has four key parts that have been identified in previous published work: 1) trained community volunteers who meet with clients in their homes and gather health and social information; 2) the use of technology for collecting and sharing information between clients, volunteers, and the health care team; 3) interprofessional primary health care teams who support clients with their health goals and needs; and 4) community engagement and connections; all of these parts encircle the client [5, 6, 35]. Based on the previous implementation and evaluation of the program, including implementer, participant, and stakeholder consultation, these have been identified as the core intervention components. The “core component” literature understands this term to mean the essential functions, principles, activities, or elements that are needed to produce the desired elements, i.e., what elements produce a potentially effective program [36]. While every community included of these key elements, there were distinctions even between some of these core components when the program was adopted and subsequently adapted by each of the six community. It is important to understand context differences when an intervention is implemented in a new setting [37]. Based on the qualitative matrix exercise, there were distinct differences between communities in the areas of interprofessional teams, the volunteer program, and the client experience. Beyond deepening our understanding of the core components of Health TAPESTRY that had distinctions between communities, we also structured this paper including the first two domains of the Model for Adaptation Design and Impact (MADI): 1) adaptation characteristics; and 2) possible mediating or moderating factors, which we include thoughts about in the Discussion [37]. The third domain of MADI is implementation and intervention outcomes, but this evaluation was not designed to identify or compare outcomes [37].

## Results

### Individual participants

In total 135 people participated in either an interview or a focus group. No one explicitly refused to participate or withdrew from the study. Focus groups were about one hour long and interviews were approximately 30 min. Our sample had representation from all participant groups from each of the six communities (Table 1) and our sample size per site aligns with the expected number of people needed to meet saturation based on the previous implementation of Health TAPESTRY.

**Table 1:** Individuals who participated in this evaluation and in the program overall

Participant Category	Community ***N participated in evaluation (N involved in program)***						
	A	B	C	D	E	F	Total
Clients	7 (132)	8 (223)	6 (84)	6 (48)	4 (30)	8 (50)	39 (567)
Clinical Managers	2 (2)	2 (2)	1 (1)	1 (1)	1 (1)	1 (1)	8 (8)
Health Care Providers & Other Staff	10 (16)	15 (40)	7 (8)	10 (17)	7 (10)	10 (13)	59 (104)
Volunteer Coordinators	1 (1)	1 (1)	0* (0*)	1 (1)	2 (2)	1* (1*)	6 (6)
Volunteers	5 (23)	4 (63)	2 (0*)	5 (45)	2 (8)	5 (37*)	23 (176)
Total	25 (174)	30 (329)	16 (93)	23 (112)	16 (51)	25 (102)	135 (861)

*The Community C/Community F Volunteer Coordinator is included in Community F, as are the volunteers as multiple volunteers overlapped across the two communities

### Description and distinction between cases: context differences and adaptation characteristics

The six communities that Health TAPESTRY was implemented in ranged from a small town to a large city. The participating FHTs differed in their roster size, number of physicians, number and assortment of allied health professionals, and the services the clinic offered. One FHT consisted of two separate clinics, each with their own huddle teams. Table 2 describes three key areas relating to implementation and the flow of information throughout the Health TAPESTRY program for each community. Table 2 also maps to Domain 1 of MADI: adaptation characteristics; this domain asks *what* was modified, the *nature* of the adaptation, *when* did the adaptation occur, for whom/what was the adaptation made (i.e., the *reason*), and *who* participated in the decision [37].

**Table 2:** Description of cases and distinctive elements

	Community A	Community B	Community C	Community D	Community E	Community F
**Community Descriptors: Context Differences**						
Community type/size^a^ *Approximate population*	County of multiple small towns *62,000*	Large city *748,000*	Small town *2700*	Small town *18,000*	Small city *78,000*	Medium city *330,000*
FHT size	36 family doctors; 47,000 patients	21 FTE family doctors; 35,000 patients	6 family doctors; 6400 patients	11 family doctors; 15,000 patients	6 family doctors; 7300 patients	5 family doctors; 6300 patients
Availability of programs and services^b^	Many programs and services available although may have to travel to other communities.	A wide range of program and services available within community.	Some programs and services available, although may have to travel to other communities.	Some programs and services available, although may have to travel to other communities.	Many programs and services available within the community.	Many program and services available within the community.
**Huddle Elements: Adaptation Characteristics**						
Huddle size (n; of n disciplines)	5; of 5 disciplines	Site 1: 8; of 8 disciplines Site 2: 5; of 5 disciplines	4; of 3 disciplines	6; of 5 disciplines	5; of 4 disciplines	6; of 5 disciplines
Huddle lead characteristics	Registered Nurse; Existing team member	Site 1: Pharmacist; Existing team member Site 2: Registered Practical Nurse; Newly hired	Physician Assistant; Newly hired	Registered Nurse; Newly hired	Administrative Assistant; Existing team member	Registered Nurse; Newly hired
Physicians in the huddle	Physician champion often attended. Huddle lead contacted patients’ MRP separately.	Physician champion often attended. Huddle lead contacted patients’ MRP separately.	Physician champion always attended. Most often it was the patients’ MRP.	Physician champion always attended. Huddle lead contacted patients’ MRP separately.	Physician champion always attended. Most often it was the patients’ MRP.	Physician champion sometimes attended. Huddle lead contacted patients’ MRP separately.
Inclusion of a System Navigator in the huddle	No	Yes	No	Had an outreach nurse.	No	No
VC attendance in the huddle	Attended regularly	Attended when invited	Attended regularly	Attended when invited	Attended regularly	Attended regularly
Length of time in Health TAPESTRY	Less than 3 years	More than 3 years	Less than 3 years	Less than 3 years	Less than 3 years	Less than 3 years
**Volunteer Program Elements: Adaptation Characteristics**						
VC’s location	Neighbouring community	Same community	Neighbouring community	Neighbouring community	Same community	Same community
VC’s connection to the huddle	Integrated into the huddle, often contributed to care planning.	Invited to a weekly meeting outside of the huddle with the huddle leads to discuss specific cases.	Integrated into the huddle, often contributed to care planning.	Invited to participate in some (not all) huddles, connected with the huddle lead as needed.	Integrated into the huddle, often contributed to care planning.	Integrated into the huddle, often contributed to care planning.
Continued education for volunteers (i.e., Lunch ‘n’ Learns)	Topics: Elder abuse, Dementia, Goal setting, interview/note taking skills, emergency preparedness, Advanced care planning. Also allowed volunteers to share experiences and problem-solve.	Topics: Dementia, System navigation, goal setting, advanced care planning. Also provided program updates and had group discussions.	Topics: TAP-App, Goal setting, Advanced care planning. Also allowed volunteers to share experiences and problem-solve.	Topics: Dementia, COPD, Goal setting Also provided program updates and had group discussion.	No lunch ‘n’ learns. Initial classroom training had extra module on community programs and services. Volunteers were invited to debrief with VC after visits.	Topics: TAP-App, goal setting Also allowed volunteers to share experiences and problem-solve.
Volunteer role in community connections	Volunteers helped make connections to programs.	Volunteers did not help make many connections to programs.	Volunteers helped make connections to programs.	Volunteers did not help make many connections to programs	VC helped make connections to programs.	Huddle lead provided detailed instructions for volunteers to help make connections to programs.
**Client Experience Elements: Adaptation Characteristics**						
Mode of client recruitment	Invitation mailed to eligible patients. Follow-up phone call to those identified by MRP.	Invitation mailed to eligible patients. Follow-up phone call to those identified by MRP.	Phone call invitation to eligible patients. Mailed invitation package to interested individuals.	Phone call invitation to eligible patients. Mailed invitation package to interested individuals.	Phone call invitation to eligible patients. Mailed invitation package to interested individuals.	Invitation mailed to eligible patients. Follow-up phone call to all.
Client-friendly TAP-Report sent to each participant	Yes	Yes	Started partway through implementation.	No, contacted client by phone.	Yes	Started partway through implementation.

*COPD* Chronic obstructive pulmonary disease, *FTE* Full Time Equivalent, *FHT* Family health team, *MRP* Most responsible provider, *TAP-Report* Personalized summary of client survey responses, *VC* Volunteer coordinator, ^a^Description based on provincial census data, ^b^Based on scan of communities’ resources and on data from interviews and focus groups

The huddle, volunteer program, and client experience elements in Table 1 describe *what* was modified and the *nature* of that adaptation, along with *when* it occurred (if during implementation – unstated times occurred prior to program implementation in that community). In each community, the *reason* for each of these adaptation differences was made largely based on feasibility within their unique setting. *Who* made these decisions? The key personnel from the interprofessional primary care team made most of the decisions (including those related to their team, client communication, or volunteer connections with their team), supported by the overall Health TAPESTRY program manager who ensured the changes did not stray from the core components; the volunteer coordinator both made decisions and took action regarding volunteer training.

The following describes the saliency of the themes within the three key areas of difference we found and chose to examine: interprofessional teams’ patterns of work, volunteer program coordination, and client experience.

#### Interprofessional teams’ patterns of work

There were three key sub-categories within the larger category of interprofessional teams’ patterns of work: things that were not working well for the primary care teams, the nature of human resources at the primary care practices, and the role of physicians outside the huddle (Table 3). Most communities were in clear agreement that physician buy-in was a challenge, and that workload increased among all clinical sites to some degree. Communities that reported the most difficulty getting physician buy-in for the program noted that many physicians did not attend the huddles or contribute to discussions. A few primary care teams experienced a lack of clarity about who was responsible for coordinating follow-up care for clients.

**Table 3:**
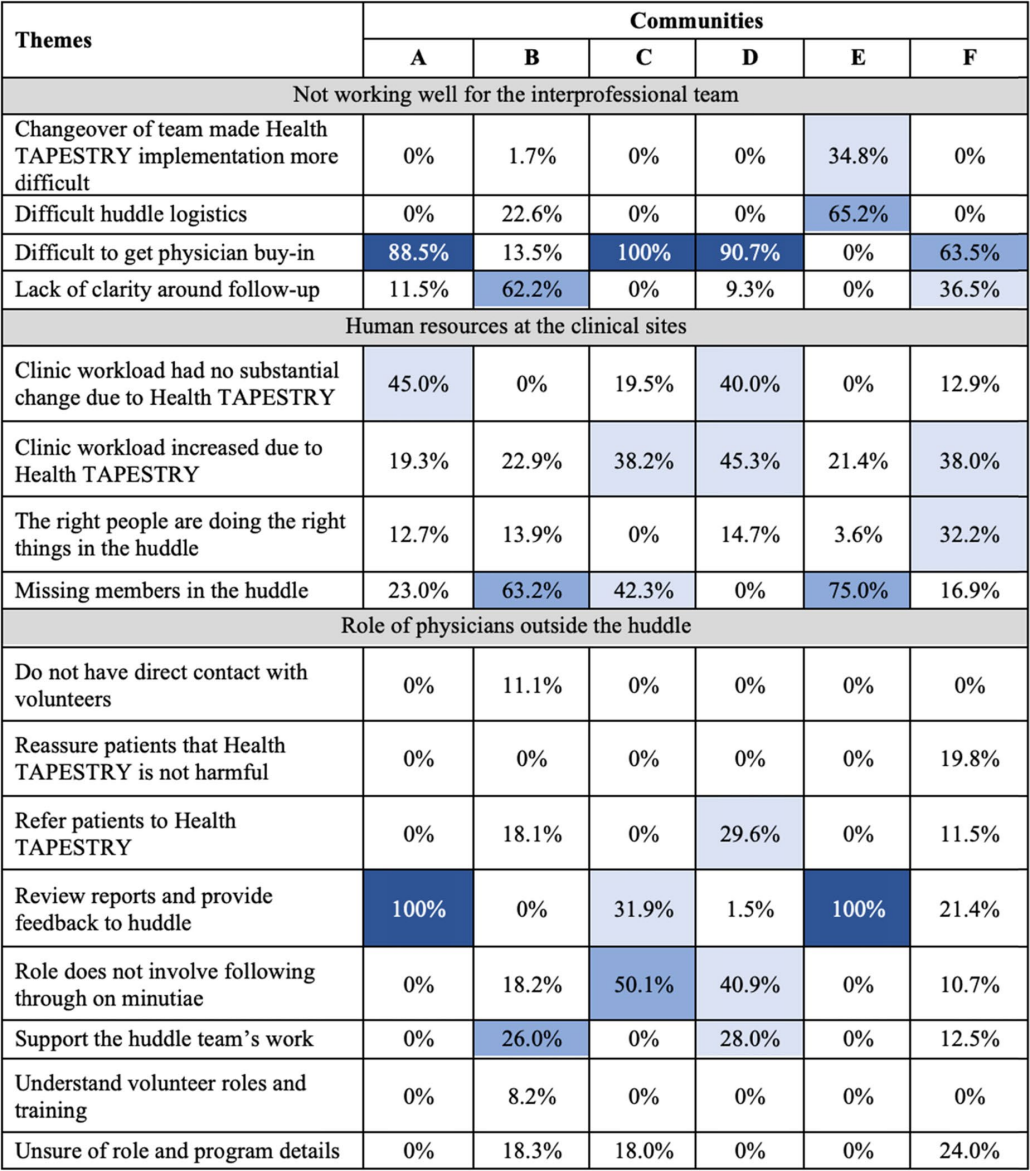
Matrices of the qualitative themes relating to interprofessional teams’ pattern of work by community

Percentages indicate the frequency that each theme was discussed within a category by community; White = 0-25%; Light blue = 26-50%; Medium blue = 51-75%; Dark blue = 76-100%

Regarding human resources, smaller huddle groups noted gaps in the composition of their huddle teams. In these clinical sites, members felt they were missing the expertise of a specific discipline which could have improved the huddle or client care coordination process, such as a social worker. A substantial portion of participants felt that the program caused no changes to their workload, although other participants reported an increase.

Perspectives on the role of physicians differed vastly in each community. In clinical sites where the physicians’ role was clear, the role was described as only including the task of reviewing reports for their patients and providing feedback to the huddle. Other clinical sites described the physicians’ role as having a broad scope, which also included supporting the huddle team’s work, and to validate and support their recommendations.

#### Volunteer program coordination

There were three main sub-categories within the volunteer program coordination category: what was working well, what was not working well, and what the volunteer coordinator role was. Overall, stakeholders across communities agreed that volunteers were well trained for client visits and the communities collectively agreed that connecting with other stakeholders in the study was part of the volunteer coordinator’s role (Table 4).

**Table 4:**
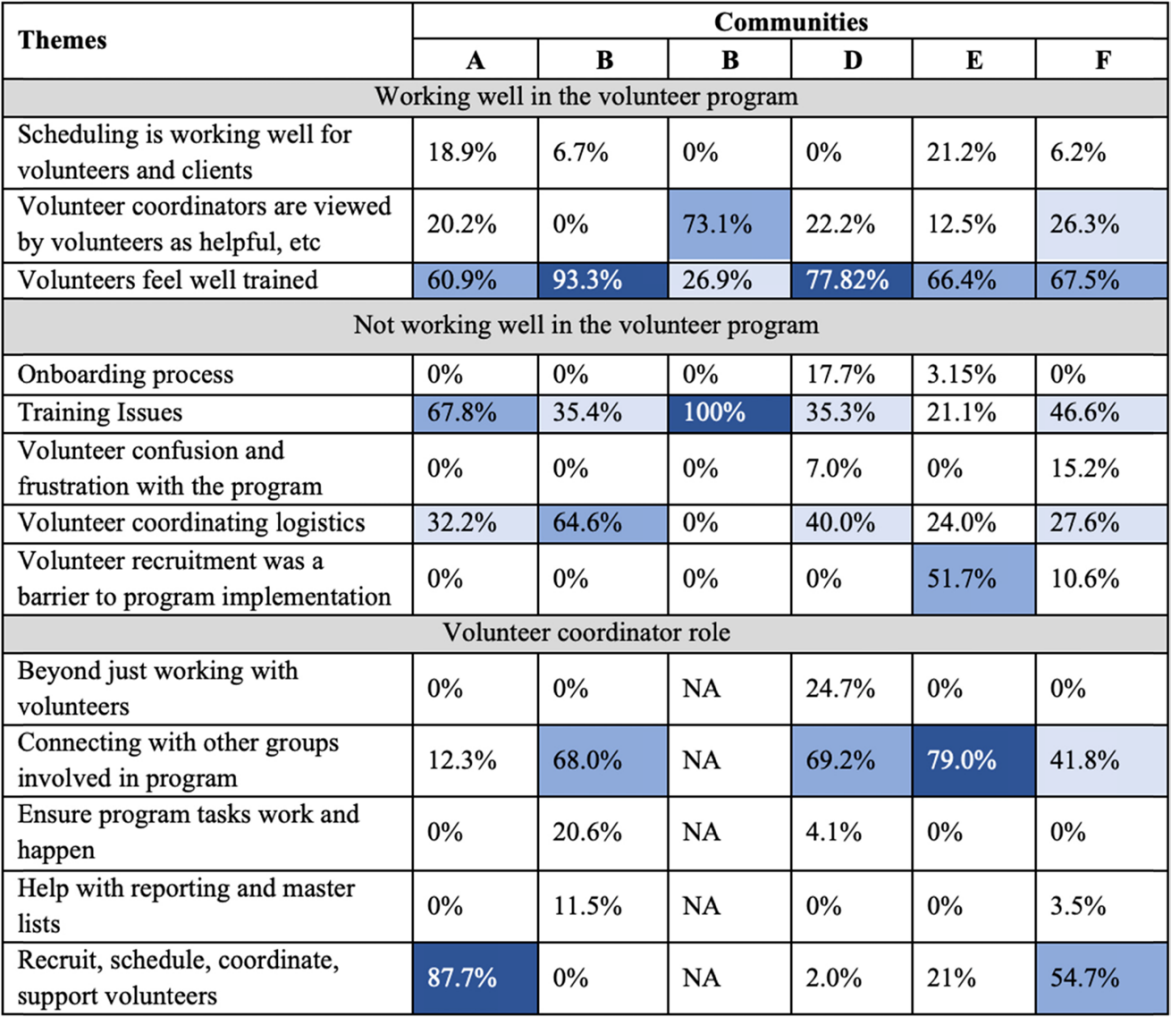
Matrices of the qualitative themes relating to volunteer coordination by community

Percentages indicate the frequency that each theme was discussed within a category by community; NA = not applicable, as all data from the Community C/F volunteer coordinator’s interview is captured under Community F; White = 0-25%; Light blue = 26-50%; Medium blue = 51-75%; Dark blue = 76-100%

Volunteers in most communities felt that they were well-trained, however half of the communities felt additional training and practice with the technology would have been helpful. The onboarding experience was particularly frustrating for volunteers in one community. Volunteer recruitment was a challenge experienced particularly by the more rural communities. There were differences in the way that the role of the Volunteer Coordinator (VC) was perceived in each community. In three communities, participants most often described this role as recruiting, scheduling, coordinating, and supporting volunteers, while in the other three, the VC role was most often described as being the liaison who connects with other groups in the program such as the FHTs, research team, or community programs.

#### Client experience

In all communities, at least some participants said that the client experience was generally positive (Table 5). Clients described benefits of the program, such as having someone to talk to, and that the program reaches some clients in need. The opposite was also true for at least some client respondents, again in all communities: that the program was reaching clients who were *not* the right fit for the program, although this was less of a common theme in Community A. Participants in all communities, but especially in Community A and F, felt that recruitment could be further targeted to those who would most benefit. The program goals and purpose were unclear to some clients. Communities reported challenges with clients getting appropriate clinical follow-up for the issues identified in TAP-Reports, although this issue varied per community.

**Table 5:**
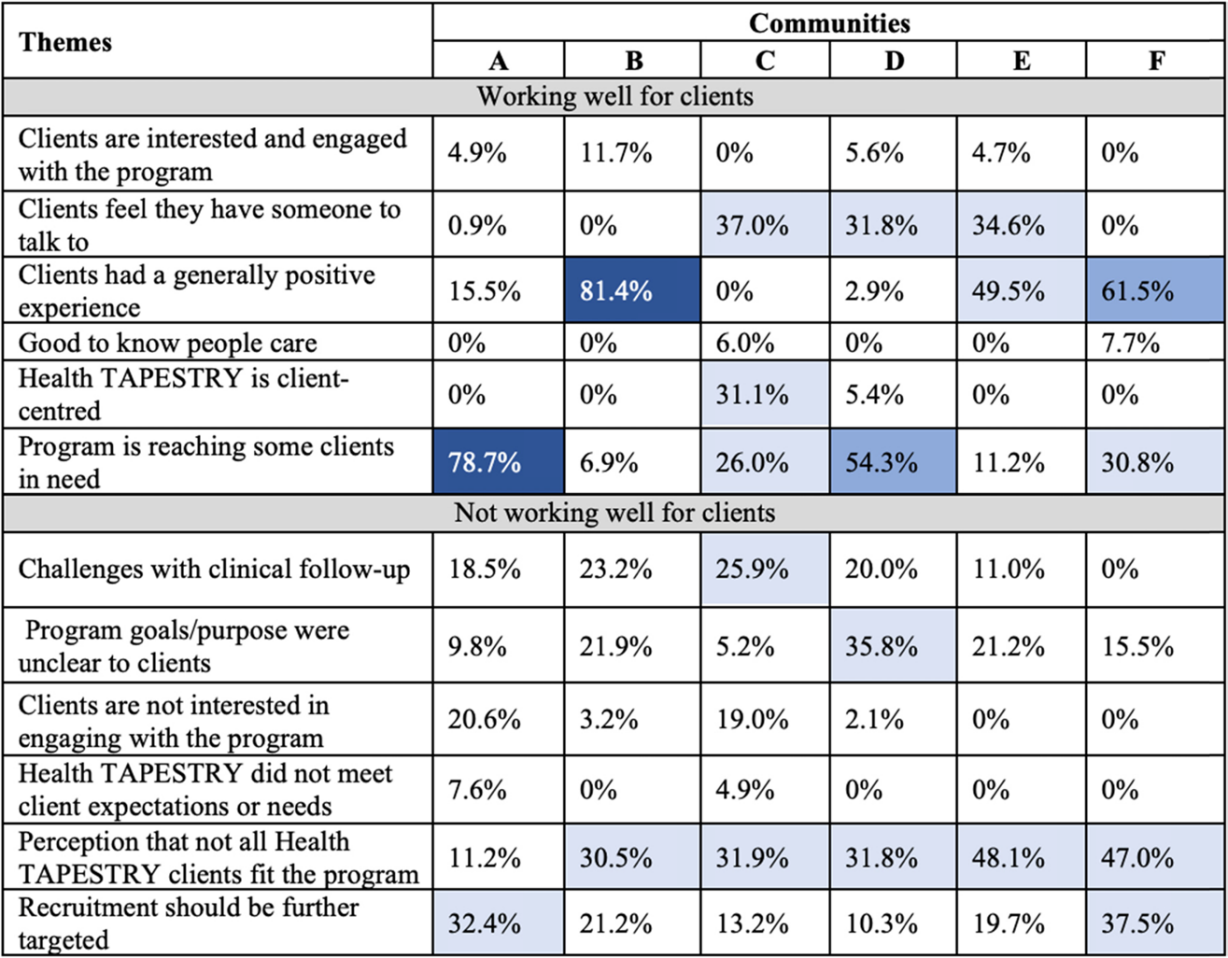
Matrices of the qualitative themes relating to client experience by community

Percentages indicate the frequency that each theme was discussed within a category by community; White = 0-25%; Light blue = 26-50%; Medium blue = 51-75%; Dark blue = 76-100%

## Discussion

The six communities that implemented Health TAPESTRY were variable in community size; primary care practice size, composition, and workflow; and general choices in program implementation. While each community was able to successfully implement Health TAPESTRY, i.e., run the program, it is clear that certain elements affected implementation more than others, in either a positive or negative sense, and those elements differed by community. These considerations of potential mediating or moderating factors include both context differences and adaptation differences, and map onto the second domain of MADI. These distinctions are discussed below.

The implementation of Health TAPESTRY by interprofessional health care teams was affected by both the availability and type of resources at each clinical site. For example, role clarity and physician buy-in were not issues raised by Community E, which had the smallest huddle. The community’s small size may have simplified task delegation and encouraged physician buy-in, leaving little room for confusion. However, small huddle size also came with challenges. For example, the two smallest huddles (Community C and E), reported they were missing key disciplines that would have been beneficial. In communities where physicians were involved beyond providing feedback on TAP-Reports, their role was less clear. For example, Community B had the highest proportional mentions of lack of clarity around follow up, and physicians’ main role was less defined in this community. This community had two clinical sites, and while one had a very active champion for Health TAPESTRY, the other did not through the duration of the program. A champion is central to interprofessional teamwork [11] and may have affected program implementation. In three of the six communities, the huddle team reported increased workload which is common when implementing new innovations [9]. The huddle leads at these communities were also newer employees and may not have had the time to develop the strong relationships and credibility with their colleagues, which are foundational to collaborative teamwork [7, 11].

Each community had distinct perceptions of the volunteer coordinator role. There seemed to be a connection between communities where more positive attributes of VCs were described and where the VCs conducted more activities beyond the role description, such as holding meetings with unstructured time for volunteers to discuss experiences and problem solve issues. Opportunities for feedback and social interaction can foster positive relationships, and contribute to volunteer satisfaction, retention, and engagement [15, 38, 39]. Further, we speculate that how the VC role was defined and described, the specific skills and experiences of both VCs and huddle leads, and the relationships between VCs and clinical teams all contributed to differences in program implementation. The integration of a VC into the huddle team may affect implementation as the fit of a person with a team is an important facilitator of collaborative teamwork [7, 10, 11].

Volunteer onboarding and recruitment issues were largely limited to a certain community each. Community D identified issues with onboarding despite having the same process as three other communities. This community already had a vibrant volunteer group; the volunteers were well-accustomed to volunteering so may have had unique preferences and expectations that differed from those in other communities. Community E had a particularly hard time recruiting volunteers. It was not the smallest community implementing Health TAPESTRY, although it may be considered more remote; transportation, given the spread of this community, was one of the main impediments to volunteer recruitment.

Regarding client experience, participants in Community C and D mentioned less often that clients had a “generally positive experience” compared to other communities. While there are likely many explanations for this finding that may not be captured in our analysis, one possibility is that these two communities did not consistently use the client-friendly TAP-Report. Providing clients with information about the proposed plan of care facilitates patient-centered communication and has the potential to better include them in decision making about their own health and health care, which can lead to increased patient satisfaction [40]. The clients in Community D also were more likely to describe a lack of clarity about Health TAPESTRY compared to other communities. Clients at this community were also invited to participate in Health TAPESTRY via a phone call followed by a letter, whereas other communities recruited by a letter followed by a phone call. Being able to refer to a written explanation of Health TAPESTRY while discussing the program over the phone may have been beneficial for clarity.

One strength of this study was the large sample size and the inclusion of multiple stakeholders which provided us with a comprehensive dataset about each community. Another strength was our use of matrices to identify patterns within and between communities from qualitative data which can be challenging given the size of our sample. One limitation of the study was that the data collected only represented a subset of all the clients, volunteers, primary care team members, and others involved in the study. Only this subset was included due to both consent and feasibility: participation in the evaluation was optional and there were a great number of individuals involved in the program. Further, the participants all self-selected to participate which may have led to a participation bias.

## Conclusions

Through our comparative case study, we were able to explore the differences between six communities implementing Health TAPESTRY. Despite all communities having the same key program elements including an interprofessional primary care team and trained community volunteers, the program as implemented on a community-by-community basis did differ. The size and spread of communities and previous engagement in volunteering affected volunteer recruitment and onboarding. The size of FHTs also affected implementation: small FHTs may have had fewer resources, but also more role clarity. Better linkages between the volunteer program and the interprofessional team seemed to produce more positive perspectives. Based on our learnings in this study, we have several key suggestions for future similar program implementation: 1) provide clear and defined roles for every stakeholder in the implementation program; 2) engage all stakeholder in all relevant elements from the beginning to build buy-in (for example, with physicians in our study); 3) target the clients who should benefit the most from the intervention with specific recruitment efforts; 4) prepare for differences in implementation based on community size or remoteness (e.g., know from the start that volunteer recruitment may be more difficult and may take longer in smaller, more rural communities); 5) prepare for differences in implementation based on clinic size or resources (e.g., know that smaller clinics with fewer allied health professionals will likely have gaps). Overall, as with any community-based intervention, context is key.

## Supplementary Information

**Additional file 1.** Interview guide used for huddle members, health care providers, volunteers, and clients.

**Additional file 2.** Completed COREQ checklist used for reporting studies with qualitative data.

## Data Availability

The datasets analysed during the current study are not publicly available since they contain identifying information, but are available from the corresponding author on reasonable request.

## Abbreviations

CCS: Comparative case study
FHT: Family health team
VC: Volunteer coordinator
MRP: Most responsible provider
