# Supplementary material for: Understanding how context and culture in six communities can shape implementation of a complex intervention: a comparative case study
Source: BMC Health Serv Res. 2022 Feb 17;22:221. doi: 10.1186/s12913-022-07615-0 (PMC8855589; doi:10.1186/s12913-022-07615-0)
Supplement: Supplementary file 1 — Additional file 1. Interview guide used for huddle members, health care providers, volunteers, and clients. [file 12913_2022_7615_MOESM1_ESM.pdf]

Additional File 1. Interview and Focus Group Guides
Volunteers

I. OVERALL UNDERSTANDING OF HEALTH, FACILITATORS & BARRIERS

1. What do you think is the goal or purpose of the Health TAPESTRY program?

In answering the next set of questions, think about all of the components of Health TAPESTRY including the use of volunteers, technology, interprofessional teams, community connections, and system navigation to health and social services:

2. Thinking of your experiences in Health TAPESTRY overall, what are the things that are working well ? *General Probes:* What are the facilitators? What are the strengths? *Specific Probes:* Think about: Onboarding; Interactions with the Volunteer Coordinator; Scheduling of initial and follow up visits; Home visit experience; Experience working with a volunteer partner; Supporting patients to link with other community services; Connecting with the team at the clinic/primary care team.
3. What do you think are the things that are NOT working well so far? *Probes:* What are the barriers to the success of the program? What are the weaknesses? Do you have any suggestions on how to address or overcome these challenges? *Specific Probes:* Onboarding? Interactions with the Volunteer Coordinator? Scheduling of initial and follow up visits? Home visit experience? Your experience working with a volunteer partner? Supporting patients to link with other community services? Connecting with the team at the clinic/primary care team?
4. Do you think there are any drawbacks, threats or risks in using Health TAPESTRY, and if so, what are they? *Prompts*: for you, the client or the health care team?

II. NORMALIZATION OF HEALTH TAPESTRY INTO VOLUNTEER PRACTICE

In the next set of questions, we would like to know how easy or difficult it has been to take on the role of a Health TAPESTRY volunteer.

5. How do you understand your role (e.g. tasks and responsibilities) in relation to the Health TAPESTRY program? *Prompt:* How do you understand the parameters of your role? What are the current barriers to performing your role? What do you see as limitations? Where do you lack clarity in your role? Do you have any suggestions about how to address or overcome these challenges?
6. How do you understand your role in relation to others involved in Health TAPESTRY such as the health care team, clients, and your volunteer pair? Probe: How well do you feel teamwork, collaboration, and communication worked across everyone involved in Health TAPESTRY? Do you feel the right people are doing the right things?

III. RESOURCES

In this section we would like to know what you think about the resources that have been allocated to Health TAPESTRY to support the program, in terms of financial resources, human resources, training, time, space etc.

7. How well do you feel you were trained and prepared to be a Health TAPESTRY volunteer? *Prompts:* by the VLC, by the initial in-person training launch, with support from the Volunteer Coordinator? *Probe:* Describe if and how the orientation training you have received has influenced you comfort and confidence in your volunteer role?
8. What did you think about the training? *Prompt:* on the VLC, in-person, the paper Volunteer Guide. *Probe:* Comment on any redundancies, usefulness, and or gaps.
9. How has the Health TAPESTRY program provided you with the knowledge, skills, and resources necessary to navigate or connect your clients to community resources? This includes learning about community resources and how to support patients and their family to connect and use them.

IV. IMPACTS AND OUTCOMES

In this set of questions we are interested to hear your opinions about the impacts and outcomes of the Health TAPESTRY program.

10. In what ways, if any, did Health TAPESTRY impact you?

    Probes:

    a. … In your own health and wellness?
    b. …In caring for older adults over 70 years of age?
    c. …In communicating and collaborating with an interprofessional team (health care providers, clients, volunteers)?
    d. …In your ability to connect your clients to, and helping them use, community health and social services?

11. What <u>opportunities</u> came out of your participation in Health TAPESTRY that you would not otherwise have had? *Probes:* How has Health TAPESTRY affected the number of people or places that you are connected with, in your personal or professional networks (e.g. linkages with health professionals, community resources, etc.)? *Prompt:* What about the types of people and places that you are connected with? Have these changed at all as a result of Health TAPESTRY?

V. CLOSING

12. Do you have anything else to add?

Volunteer Coordinators

I. OVERALL UNDERSTANDING OF HEALTH TAPESTRY

1. What do you think is the goal or purpose of the Health TAPESTRY program?

In answering the next set of questions, think about all of the components of Health TAPESTRY including the use of <u>volunteers</u>, <u>technology</u>, <u>interprofessional teams</u> and system <u>navigation</u> to health and social services in the community:

2. Thinking of the Health TAPESTRY intervention <u>overall,</u> what are the things that are <u>working well</u> so far with Health TAPESTRY? *General Probes:* What are the facilitators? What are the strengths? *Specific Probes:* Interactions with the Volunteers? Scheduling of initial and follow up visits? Connecting with the health care team at the clinic?
3. What do you think are the things that are NOT working well so far?
   Probes: Re: volunteers, technologies, clients, connecting to the clinic, etc? What are the barriers to the success of the program? What are the weaknesses? Do you have any suggestions on how to address or overcome these challenges?
4. How did you find the experience of scheduling volunteers in pairs? What do you think about this arrangement of volunteers working in pairs? Probe: What worked well and what did not in this arrangement?
5. Do you think there are any drawbacks, threats or risks in using Health TAPESTRY, and if so, what are they? Prompts: for you, the volunteers, the clients, or the health care team?

II. NORMALIZATION OF HEALTH TAPESTRY IN VOLUNTEER PRACTICE

In the next set of questions, we would like to know how easy or difficult it has been to take on the role of a Health TAPESTRY volunteer coordinator.

6. How do you understand your role (e.g. tasks and responsibilities) in relation to the Health TAPESTRY program? *Prompt:* How do you understand the parameters of your role? What are the current barriers to performing your role? Do you have any suggestions about how to address or overcome these challenges?
7. How do you understand your role in relation to others involved in Health TAPESTRY such as the health care team, the volunteers, and the clients? Probe: How well do you feel teamwork,

collaboration, and communication worked across everyone involved in Health TAPESTRY? Do you feel the right people are doing the right things?

III. RESOURCES

In this section we would like to know what you think about the resources that have been allocated to Health TAPESTRY to support the program, in terms of financial resources, human resources, training, time, space etc.

8. How well do you feel you were trained and prepared to be a Health TAPESTRY volunteer coordinator?
9. How has the Health TAPESTRY program provided you with the knowledge, skills, and resources necessary to aid volunteers in navigating clients to community resources?

IV. IMPACTS AND OUTCOMES

In this set of questions we are interested to hear your opinions about the impacts and outcomes of the Health TAPESTRY program.

10. In what ways, if any, did Health TAPESTRY impact you?
    Probes:
    a. …In caring for older adults over 70 years of age?
    b. …In communicating and collaborating with the interprofessional team (health care providers, clients, volunteers)?
    c. ….In your own health and wellness?

V. CLOSING

11. Do you have anything else to add?

Clients

I. OVERALL UNDERSTANDING of Health TAPESTRY

1) How would you describe the Health TAPESTRY program to others? What is its main purpose?

II. Health TAPESTRY IN PRACTICE

Next, in this set of questions, think about all the experiences you had in the Health TAPESTRY program, for example getting signed up for the program, scheduling with the volunteer coordinator, having volunteers come to your house, completing the various surveys and the goal setting exercise with volunteers, and any follow up from the team at the clinic or the volunteers.

2) Thinking of the Health TAPESTRY program overall, what are the things that are working well so far with Health TAPESTRY?

   Probes: What are the *facilitators*? What are the *strengths*?

3) What do you think are the things that are NOT working well so far?

   Probes: What are the *barriers to* success of the Health TAPESTRY program? What are the *weaknesses* of the program? Do you have any suggestions on how to address or overcome these challenges?

Now that we've talked about your experience with Health TAPESTRY, I want to ask you about a few other aspects of the Health TAPESTRY program. This includes your impressions of your healthcare team and links to community programs and services OVER THE LAST 6 MONTHS. Let's start with the healthcare team, which includes you, your healthcare providers (including your family doctor and other

staff at the clinic, such as the nurse practitioner, occupational therapist, social worker, and pharmacist), the Health TAPESTRY volunteers, and your caregivers/family members.

4) How has the Health TAPESTRY program affected your experiences communicating and working with members of your health care team, if at all?
   Probes: How has your relationship with your clinic changed since your involvement in the program, if at all? How has your level of confidence or satisfaction with healthcare at your clinic changed, or not?

5) As a result of Health TAPESTRY, were you linked or referred to any community programs or services such as home support or community groups by your volunteers or the health care team at your clinic? If so, tell me about the experience about getting linked to those programs.
   Probes:
   a) *How did you use these programs or services?*
   b) *Were you satisfied with which community resources you were referred to or were suggested to you as a result of Health TAPESTRY? How did they support your wellbeing, if at all?*
   c) *In what ways if any, do you use community based programs or services differently since you became involved with Health TAPESTRY?*

6) How would you describe your experiences of how your health care has been coordinated over the last 6 months, among you and the different members of your health care team: the volunteers, your family doctor, nurses, other health care providers, community programs or services, and any specialists that you see?

7) How would you describe the ways, if any, in which participating in the Health TAPESTRY program helped you navigate through the health care system? How could navigation through the health care system been improved?

8) What risks or drawbacks might exist from taking part in the Health TAPESTRY program, for you or other participants?

9) How has participating in Health TAPESTRY been different from the usual care you get with your clinic, if at all?
10) Thinking about your experience overall with Health TAPESTRY, in what ways, if any, did the program help you meet your health goals and needs?
   Probe: Has your awareness of health-related choices affecting your well-being changed since your involvement with the program?
11) Is there anything else you would like to add?

Huddle Members, Physicians, Primary Care Staff

I. UNDERSTANDING THE IMPLEMENTATION OF HEALTH TAPESTRY

In the first set of questions, think about all of the components of Health TAPESTRY including the use of volunteers, technology, interprofessional teams, community and social program and service connections, and system navigation throughout:

3. Thinking of the Health TAPESTRY intervention overall, what are the things that are working well so far with Health TAPESTRY? Probes: What are the facilitators? What are the strengths?

Beyond your own organization, how do any local, provincial or national factors act as facilitators for Health TAPESTRY, if at all? For example, municipal, provincial, and national strategic directions.

4. What do you think are the things that are NOT working well so far?
   Probes: What are the barriers to success of the program? What are the weaknesses of Health TAPESTRY? Beyond your own organization, how do any local, provincial or national factors act as barriers for Health TAPESTRY, if at all? Do you have any suggestions on how to address or overcome these challenges?

5. Do you think there are any drawbacks, threats or risks in using Health TAPESTRY, and if so, what are they and for whom?

II. NORMALIZATION OF HEALTH TAPESTRY INTO PRACTICE

In the next set of questions, I/we would like to know how easy or difficult it has been to integrate Health TAPESTRY with the work that you do, any ways that your work has changed as a result, and what you think of these changes.

4. What do you think were the reasons your clinic got involved in Health TAPESTRY and what do you understand or believe is your individual contribution to support it?
5. How does the Health TAPESTRY approach differ from your usual way of working as a team to support/care for older adults over 70 years of age? (prompt: What are changes in your role / responsibilities and roles of other team members as a result of TAPESTRY)
6. How do you understand your role in Health TAPESTRY in relation to other team members (including health care providers, volunteers, and clients)? PROBE: How do you understand the relationship and connections between MRPs and huddle members in the context of Health TAPESTRY?

III. IMPACTS AND OUTCOMES

In this set of questions we are interested to hear your opinions about the impacts and outcomes of the Health TAPESTRY program, as you see them.

8. * In what ways, if any, does Health TAPESTRY change the way you work on a daily basis?
9. How has the Health TAPESTRY program affected communication, collaboration, and teamwork across the health care team (including health care providers, clients, and volunteers), if at all?

W*e* define system navigation as "an individual or team engaging in specific activities, including: facilitating access to health-related programs and services for patients/families and caregivers; promoting and facilitating continuity of care; identifying and removing barriers to care; and effective and efficient use of the health care system for both patients/families, caregivers, and practitioners" (Valaitis et al., 2017)

10. How has the Health TAPESTRY program affected you and your team's practice of system navigation within and outside of the health care section, if at all? PROBE: How has Health TAPESTRY affected the coordination of services for your patients?
11. * What <u>opportunities</u> came out of your participation in Health TAPESTRY that you would not otherwise have had?
12. How successful is the Health TAPESTRY program in meeting the health goals and needs of the older adult clients? PROBE: In what ways will Health TAPESTRY meet their needs? What barriers do clients face in participating in the intervention?

IV. RESOURCES

In this section we would like to know what you think about the resources that have been allocated to Health TAPESTRY to support the program, in terms of financial resources, human resources, training, time, space etc. and how these are allocated across the "pillars."

13. What kinds of resources have been allocated to support Health TAPESTRY and what do you think of this? PROBE: What kind of local, state, or national performance measures, policies, regulations
14. Are the right people doing the right things in terms of skills, disciplines, training? Is anything or anyone missing?
15. Do you feel like you have the knowledge and skills necessary to effectively do the work that Health TAPESTRY requires?
    a. What about use of the technological components of the Health TAPESTRY program (e.g., the TAP-App)?
    b. What about navigating your clients through health and social systems?

V. CONTINUATION OF HEALTH TAPESTRY

16. Do you see value in the program so far – why or why not?
17. Do you have any interest in continuing your work with Health TAPESTRY after this program is complete – why or why not?
18. We would like to share a summary of the focus group highlights with the huddle as a way for you to consider any refinements in the process. How would you like this summary to be shared?

VI. CLOSING

19. Do you have anything else to add?
